# Portomesenteric Venous System Gas after CT Colonography: A Case Report

**DOI:** 10.1155/2012/420901

**Published:** 2012-09-20

**Authors:** Ilaria Sansoni, Claudia Lucia Piccolo, Ilenia Di Giampietro, Matteo Polacco, Bruno Beomonte Zobel

**Affiliations:** Complex Operative Unit of Diagnostic Imaging, Campus Bio-Medico University Hospital, Alvaro del Portillo 200, 00128 Rome, Italy

## Abstract

Portomesenteric vein gas can occur owing to a variety of interraleted factors such as loss of mucosal integrity and intraluminal overpressure, and the most common and serious cause is bowel ischemia, which requires urgent laparotomy. Nevertheless, when portal venous gas is caused by nonischemic causes, surgery is not required and it can be treated conservatively. So, its features should be carefully evaluated at CT scan, together with clinical findings. The authors report a case of an old male with portomesenteric venous system gas after CT colonography, without evidence of pneumatosis intestinalis or colonic perforation. A CT scan without enema was required after 24 hours in absence of worsened patient conditions, revealing the disappearance of gas in mesenteric vein and in the portal venous system.

## 1. Introduction

CT colonography (virtual colonoscopy, CTC) is a minimally invasive examination which could be currently considered as a possible alternative to conventional colonoscopy as a screening test for colorectal cancer [1].

This procedure requires an adequate colonic distension, which is achieved via a small flexible rectal catheter by either manual insufflation of room air or automated carbon dioxide delivery.

The reported symptomatic complication rates at optical colonoscopy are higher than those for virtual colonoscopy. The most common complication of optical colonoscopy is bowel perforation, which is reported as at an approximate rate of 0.06%–0,019% or one of 200–1300 patients in studies with large series of patients [2].

Procedural risks related with CTC need to be assessed in order to perform an adequate risk-benefit analysis and preventing complications [3].

We are now showing a case report of portomesenteric venous system gas as a benign complication of CTC following room air colonic distension.

## 2. Case Report

A 82-year-old man was admitted to our hospital with a 7-day history of fever of unknown origin. He had a history of colonic diverticulitis but no other infective hotbeds were found.

Gastrointestinal investigations were inconclusive. A virtual colonoscopy was asked in order to discover a possible cause of fever as patient could not perform the optical one because he had a very low FE and he was having oral anti-coagulant therapy.

In order to obtain a suitable bowel cleaning, patient was asked to follow a low dross content diet during the three days before the examination; the morning of the CTC, patient was asked to drink about 200 mL of dimeglumine diatrizoate (Gastrografin, Bayer-Schering) in order to tag both feces and fluid remains.

**Figure 1:**
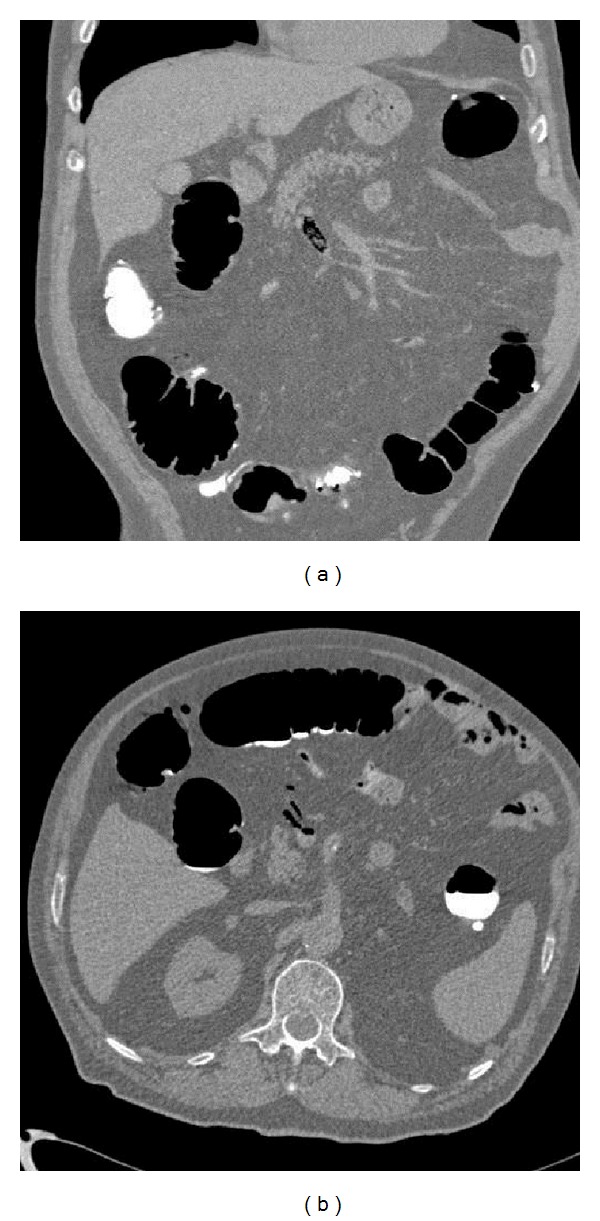
(a) Coronal contrast enhanced CT of the abdomen demonstrated the presence of gas in mesenteric vein and in the portal venous system. (b) Axial contrast enhanced CT of the abdomen demonstrated the presence of gas in mesenteric vein and in the portal venous system.

**Figure 2:**
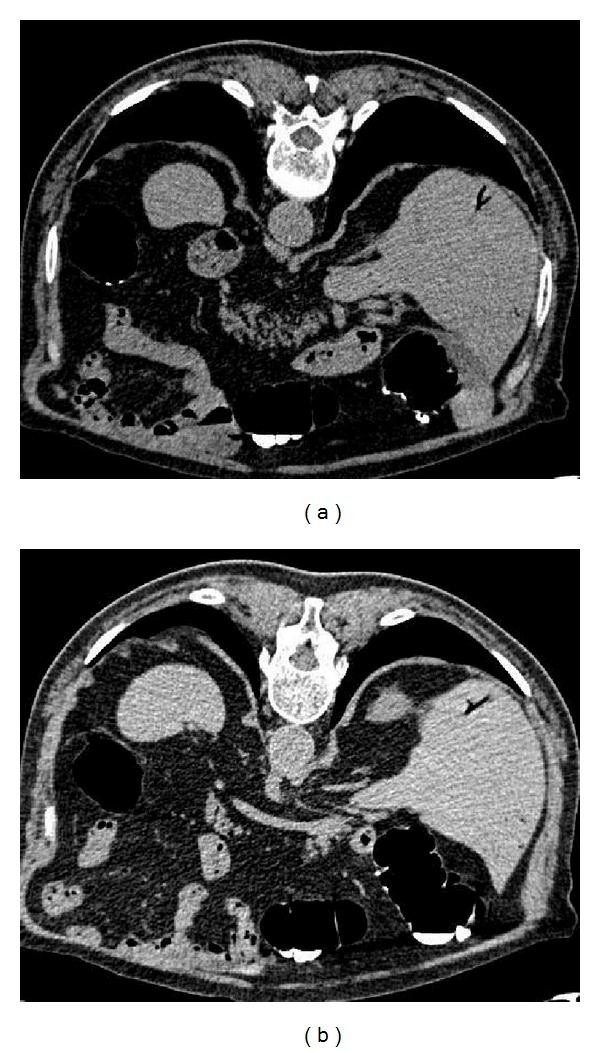
(a) Transverse CT scans revealed the presence of gas in both hepatic lobes. (b) Transverse CT scans revealed the presence of gas in both hepatic lobes.

A previous basal CT scan was obtained in order to exclude the presence of radiological sings of an active diverticulitis which is a contraindication to CTC because at risk for colonic perforation. CTC, performed after gently colonic room air distension, showed the presence of many diverticula spread on the whole colon, especially at the level of sigma, without any lumen stenosis, any evidence of masses or polyps. As extracolonic findings, it was reported the appearance of gas in mesenteric vein (Figures 1(a) and 1(b)) and within the portal venous system, involving both hepatic lobes (Figure 2).

There was no evidence of pneumatosis intestinalis or colonic perforation or free fluid in the abdomen.

Then, a CT scan without enema was required after 24 hours in absence of worsened patient conditions.

During the night patient only complained for slight abdominal pain but his conditions remained stable.

**Figure 3:**
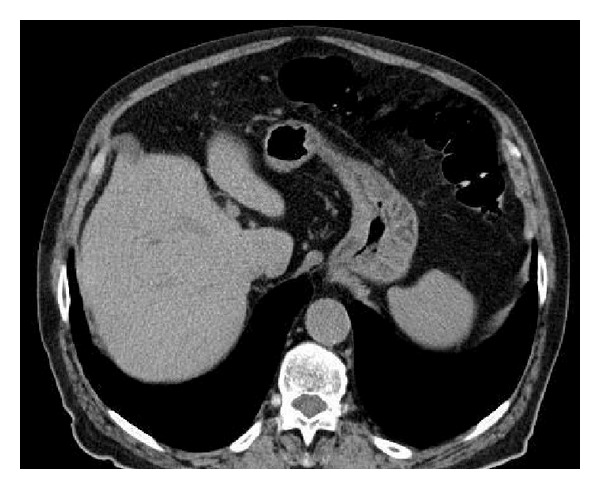
Transverse CT image showed the complete disappearance of gas in mesenteric vein and portal venous system, 24 hours after the CTC.

The CT scan performed after 24 hours revealed the disappearance of gas in mesenteric vein and portal venous system (Figure 3).

## 3. Discussion

Colonic perforation is a recognized complication of optical colonoscopy and it is reported to a rate of 0.06%–0.19%; therefore in patients at high risk, optical colonoscopy is not usually performed and is replaced by CTC [2, 4].

Until now, in literature three surveys are reported about the complication rate of CTC, related to data of Working Group on Virtual Colonoscopy (WGVC) [5], in Great Britain [6] and in Israel [3].

WGVC experience [5] reported a global rate of complications of 0,02%, with a total perforation rate of 0,009% and a perforation rate in symptomatic patients of 0,005%. In this survey two major complications occurred, that is two cases of acute renal failure related to laxative used for bowel preparation and one case of thoracic pain, in which myocardial infarction was excluded.

In the survey carried out in Great Britain [6], over 17.067 CV, 9 cases of perforation occurred, with an overall perforation rate of 0.05%, of which 2 cases had a iatrogenic cause, 2 cases were related to other concomitant diseases, 1 case to bowel preparation in a patient with multiple comorbidities.

In the Israeli survey [3], over 11.087 CTC, 7 cases of perforation were encountered, with a total rate of 0,059%. 

Until now, no deaths CTC related has been described [7].

Portomesenteric vein gas is a very rare and potentially severe radiological finding. It may occur owing to a variety of inter-raleted factors such as loss of mucosal integrity and intraluminal overpressure. The most common and serious cause is bowel ischemia, which requires urgent laparotomy, although the association of portomesenteric vein gas and this disease does not imply a worse prognosis [8, 9].

In most of cases, when portal venous gas is caused by nonischemic factors or it is secondary to invasive procedure, surgery is not required and it can be often treated conservatively [9].

Therefore, portomesenteric vein gas features should be carefully evaluated at CT scan, together with clinical findings, in order to perform the better decision regarding diagnosis and therapy.

From our experience, as portomesenteric gas following CTC was paucisymptomatic and in 24 h followup disappeared, it can be considered a benign self-limited finding, although an immediate clinical followup to confirm the absence of symptoms is recommended. Therefore, at imaging this finding should not be confused as a sign of blunt perforation.
